# Mandibular advancement impairs swallowing ability more than head extension but less than mouth opening in the supine position

**DOI:** 10.1038/s41598-019-56843-8

**Published:** 2019-12-27

**Authors:** Hiroshi Hanamoto, Eriko Togawa, Hiroharu Maegawa, Chizuko Yokoe, Mika Inoue, Aiko Oyamaguchi, Chiho Kudo, Hitoshi Niwa

**Affiliations:** 1 Department of Dental Anesthesiology, Osaka University Graduate School of Dentistry, Suita, Japan; 2 Department of Anesthesiology, Shiga General Hospital, Moriyama, Japan

**Keywords:** Dental anaesthesia, Special care dentistry

## Abstract

Mandibular advancement in the supine position may influence swallowing during dental treatment under intravenous sedation. This study investigated the influence of mandibular advancement in the supine position on swallowing ability, compared with head extension and mouth opening. The water swallowing test was performed in 13 healthy, awake, supine, adult subjects under four head and mandibular positions. An electromyogram of the suprahyoid muscles was recorded; the duration and peak amplitude were examined. A greater volume of water remained in the mouth during mouth opening and mandibular advancement relative to the neutral position; the volume in the mandibular advancement position was larger and smaller than that in the head extension position and during mouth opening, respectively. The duration of the electromyogram in the head extension position was longer than that in the mandibular advancement position, without differences in the amplitude. Thus, swallowing ability in the supine position was more impaired with mandibular advancement, relative to neutral and head extension positions, but less than that observed with mouth opening. Although unconfirmed by electromyogram, our findings suggest that head extension might improve airway patency by reducing the impairment of swallowing ability compared with mandibular advancement.

## Introduction

During dental treatment, the intra-oral fluid, including injected water, saliva, and blood, is usually removed by suction. However, the continuous and complete removal of the intra-oral fluid is difficult, resulting in the swallowing of the residual fluid. Swallowing transports the intra-oral fluid through the pharynx and to the oesophagus, and simultaneously protects the airway from aspiration by the reflex closure of the glottis^1,2^. If swallowing cannot be achieved, the fluid irritates the vocal cords and reflex coughing occurs. The cough reflex also prevents aspiration, and as a result, prevents pneumonia. Thus, both swallowing and coughing are very important mechanisms for aspiration prevention.

Although the cough reflex rarely occurs during dental treatment in wakeful patients, it sometimes occurs in patients under intravenous sedation^3^. In a previous study, we reported that the cough reflex was observed in 66% of the patients during dental implant surgery under intravenous sedation^4^. The cough reflex that occurs during dental treatment is typically accompanied by head and body movement that temporarily interrupts the dental procedure. Furthermore, sudden coughing may result in accidental injury by the rotating instruments to the non-target tissues. However, the occurrence of the cough reflex cannot be predicted, whereas airway obstruction can be prevented and improved by early detection of paradoxical breathing, the sound of snoring, and a decrease in arterial oxygen saturation.

Thus, the cough reflex is a difficult problem to manage during dental treatment under intravenous sedation. Therefore, it is important to investigate the effect of various head and mandibular positions on the swallowing ability during dental treatment in the supine position. We previously reported that both head extension and mouth opening impaired swallowing ability in the supine position^5^. During intravenous sedation, head extension and mandibular advancement are techniques sometimes used to improve airway obstruction induced by sedatives. We hypothesised that the mandibular advancement may influence swallowing ability and may be associated with the cough reflex. The objective of this study was to investigate the impairing effect of mandibular advancement on swallowing ability in the supine position as compared to the effects of head extension and mouth opening positions.

## Methods

### Study design

The investigators designed and implemented this volunteer crossover study to evaluate the effect of mandibular advancement on the ability to swallow water, and the corresponding suprahyoid (SH) muscle activity, in participants in the supine position as compared with that of the head extension and mouth opening positions. This study protocol was approved in March 2015 by the institutional review board of Osaka University Graduate School of Dentistry (protocol H26-E49). This study was registered with the UMIN Clinical Trials Registry (UMIN000021483; The effects of head extension, mouth opening, and jaw thrust on the ability to swallow in the supine position; 15/03/2016) in accordance with the Declaration of Helsinki.

### Subjects

All the subjects were healthy volunteers aged 20 to 50 years with normal dentition and American Society of Anesthesiologists physical status I. The exclusion criteria were as follows: a history of stomatognathic disorders or velopharyngeal insufficiency; palatal, pharyngeal, or laryngeal surgery; neuromuscular diseases; and speech or swallowing impairment. Participants with maxillary or mandibular prognathism, trismus (interincisal distance in the maximum mouth opening of less than 30 mm), or apertognathia were also excluded. The purpose and methods of the study were explained to each subject, and written informed consent was obtained from all subjects prior to participation.

### Recordings

The recording system and methods used in this study were similar to those described in our previous study^5^. Subjects were placed in the supine position on an operating table with an adjustable headrest (AlphaStar Top 1132.17; Maquet, Rastatt, Germany). The trunk and the extremities of each subject were maintained in a horizontal position. The subjects’ skin was cleansed with alcohol, and a pair of adhesive Ag/AgCl electrodes was placed on both sides of the midline under the chin^6^. A reference electrode was placed on the left ear. An electromyogram (EMG) of the SH muscles was recorded.

The EMG signals were amplified (ML-132, AD Instruments Japan Inc; Nagoya, Japan), filtered (below 10 Hz), fully rectified, and integrated with a time constant of 0.1 s. The laryngeal movements during swallowing were recorded with a piezoelectric pulse transducer (MLT-1010, AD Instruments Japan; Nagoya, Japan) attached to the skin above the thyroid cartilage. The information on the EMG signals and the laryngeal movements (piezoelectric sensor recordings) were stored (digital sampling rate, 10,000 Hz) on a data recorder (PowerLab 8SP, AD Instruments Japan; Nagoya, Japan) for later analysis. Moreover, a high-definition video camera (HC-V600M, Panasonic; Osaka, Japan) was used to monitor the movements of the larynx.

### Criteria for determining the head and mandibular positions

**Figure 1:**
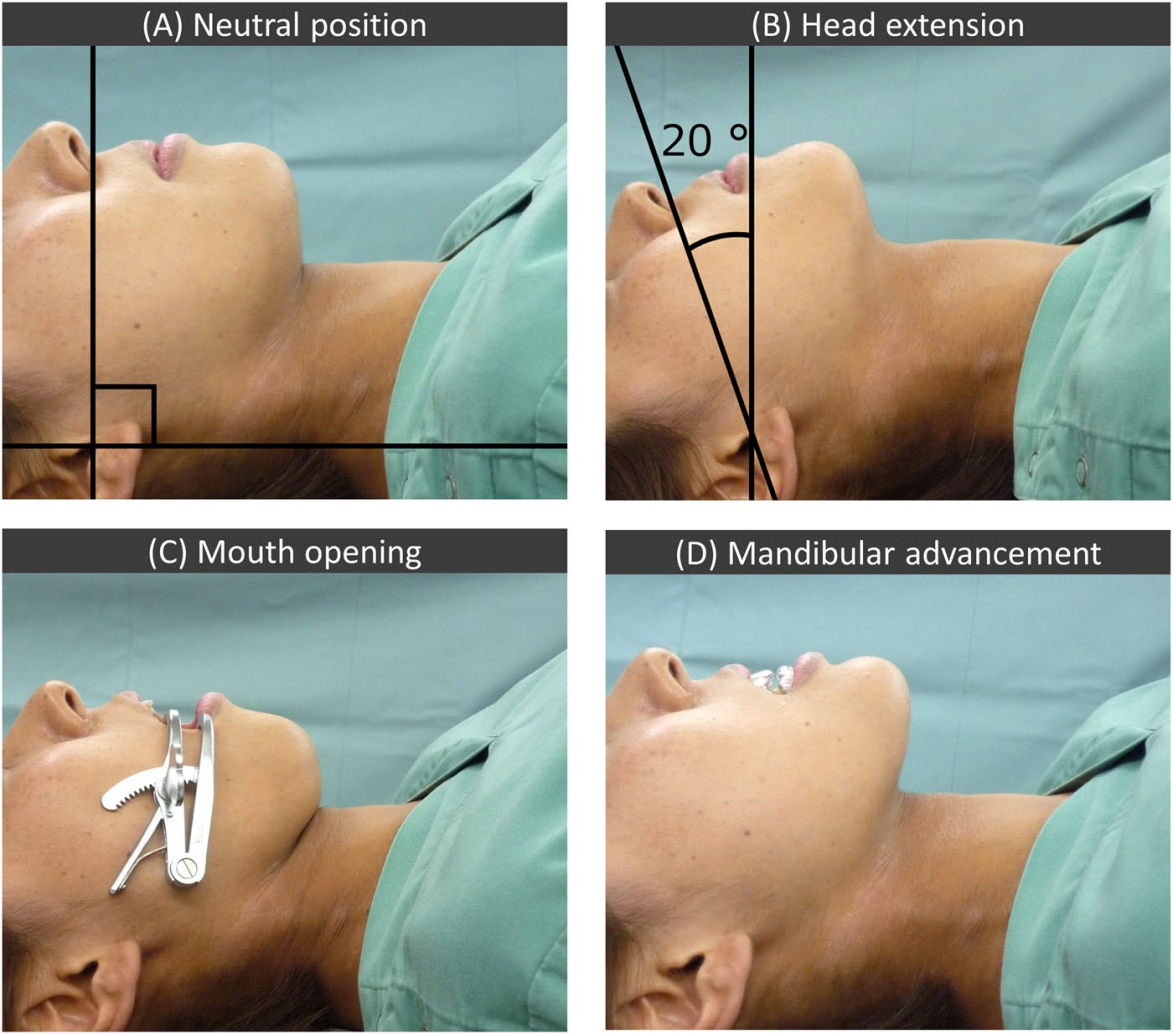
The four head and mandibular positions. (**A**) Neutral position: the ala-tragus line is perpendicular to the floor and the operating table. (**B**) Head extension position: 20-degree head extension from the neutral position. (**C**) Mouth-open position: 30 mm of interincisal distance in the neutral head position. (**D**) Mandibular advancement position: maximum mandibular advancement using a custom-made mandibular advancement appliance in the neutral head position.

We defined four head and mandibular positions as follows: neutral, head extension, mandibular advancement, and mouth opening, in the supine position (Fig. 1). ‘Neutral’ position was defined as the position in which the ala-tragus line (Camper’s line) was parallel to the line perpendicular to the floor and operating table. The ala-tragus line^7^, which extends from the inferior border of the ala to the tragus, is thought to be approximately parallel to the occlusal plane. ‘Head extension’ was defined as a 20-degree head extension from the neutral position; the angle and parallelism of the lines were confirmed using a device made of transparent acrylic that was marked with a perpendicular line, and lines at 10-degree intervals to this line^5^. ‘Mandibular advancement’ was determined as maximum mandibular advancement achieved using a custom-made mandibular advancement appliance in the neutral head position. ‘Mouth opening’ was defined as an interincisal distance of 30 mm in the neutral head position; a universal mouth opener was placed between the maxillary and the mandibular premolar teeth. The interincisal distance was measured using a slide gauge.

### Mandibular advancement appliance

**Figure 2:**
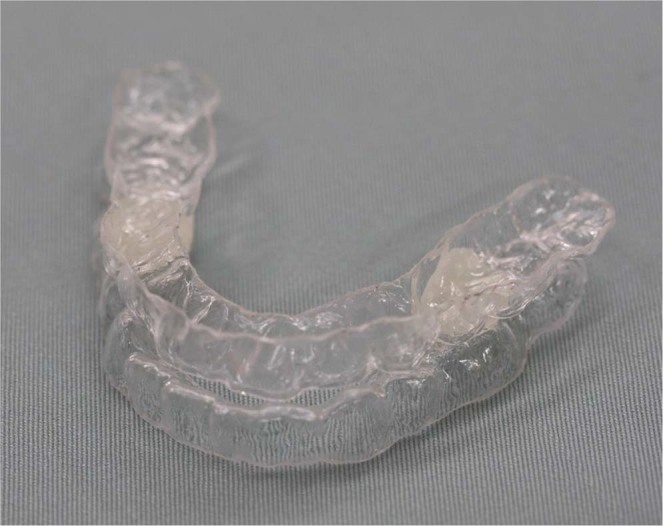
Mandibular advancement appliance. The maxillary and mandibular dental casts were made after the alginate impressions were obtained. The dental arches of both jaws were fabricated by placing a 1-mm thermoforming polyethylene plate on the dental cast using a full pressure dental thermoforming machine. The maxillary and mandibular components were joined by autopolymer resin with the maximum mandibular protrusion tolerated without discomfort or pain.

The maxillary and the mandibular dental casts were made after alginate impressions were obtained. The dental arches of both the jaws were fabricated by placing a 1-mm thermoforming polyethylene plate (Erkodur, Erkodent Erich Kopp GmbH, Pfalzgrafenweiler, Germany) on the dental cast using a full pressure dental thermoforming machine (ERKOPRESS-200 E, Erkodent Erich Kopp GmbH, Pfalzgrafenweiler, Germany). The maxillary and the mandibular components were joined using an autopolymer resin (UNIFAST III, GC, Tokyo, Japan), with the maximum mandibular protrusion tolerable without discomfort or pain (Fig. 2).

### Water swallowing test

After suctioning and removing the basal intra-oral fluid using a 6 Fr catheter, we injected 10 ml of water into each subject’s mouth, a volume used in our previous study^5^. To accurately measure the volume of the residual water, the weight was measured to one decimal place. We asked the subjects to hold the water in their mouths for 5 s and then, to swallow as much water as possible in a single attempt. After swallowing, the residual intra-oral water was suctioned using a 6 Fr catheter. The volume of the residual water was then measured by weight. The test was performed under all the four head and mandibular positions. HH randomised the order of the head and mandibular positions to four patterns, using simple randomisation with computer-generated random numbers without blocking, and announced the order after participant registration. ET enrolled and assigned participants to the interventions. This was an open trial without blinding because of the nature of the intervention. An EMG of the SH muscles was also measured during the water swallowing test.

### Study variables

**Figure 3:**
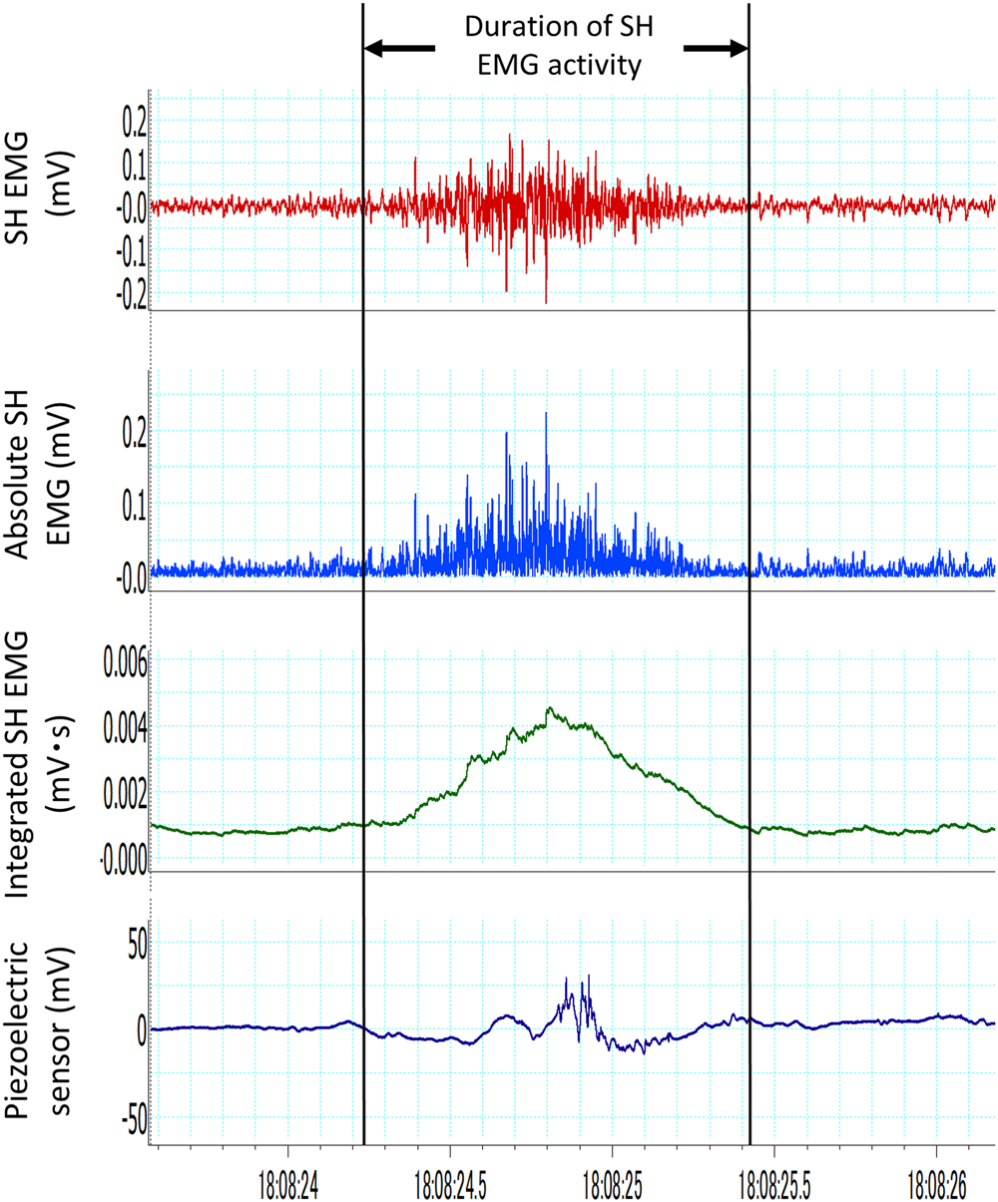
Representative electroencephalogram of suprahyoid muscles (SH EMG) and piezoelectric sensor trajectory. The duration of SH EMG activity was determined using SH EMG, integrated SH EMG, the trajectory of the piezoelectric sensor, and a video-movie. The piezoelectric sensor was used to determine the duration, defined as the time between the first negative deflection and the return to the pre-swallowing level in the trajectory. Peak amplitude was determined as the maximum absolute value of the amplitude of SH EMG. A supplementary video was also used to determine the duration.

In this study, the primary predictor variables were the four head and mandibular positions. The primary outcome variable was the volume of residual water. The secondary outcome variables were the duration of SH muscle activity and peak amplitude of the SH EMG. The duration was determined using SH EMG, integrated SH EMG, the trajectory of the piezoelectric sensor, and a video-movie. The piezoelectric sensor was used to determine the duration, defined as the time between the first negative deflection and the return to the pre-swallowing level in the trajectory^8^. Peak amplitude was determined as the maximum absolute value of the amplitude of SH EMG (Fig. 3). To normalise the peak amplitude, the value in the neutral position was determined as 100%. Both raw and normalised values of the peak amplitude were analysed. A supplementary video was also used to determine the duration of SH muscle activity^5^.

### Statistical analysis

As there are no similar, previously reported studies that evaluate the swallowing ability in the mandibular advancement position, a power analysis was not performed for this study. Therefore, we planned this study to include 13 subjects from our previous study^5^ to reduce the number of volunteers. All the statistical analyses were performed using SPSS Statistics version 22.0 (IBM Corp.; Armonk, NY, USA). Data are expressed as mean ± standard deviation. The outcome variables were analysed by repeated measures analysis of variance. Mauchly’s sphericity test was used to confirm the sphericity of the data; in cases with sphericity violation, the data were corrected using the Greenhouse–Geisser method. Intergroup comparisons were performed using Tukey’s test. A *P* value of <0.05 was considered statistically significant.

## Results

Thirteen female subjects with no routine medication were included in the complete analysis. The characteristics of the subjects are shown in Table 1. There were no subjects with macroglossia. The angle classifications of the participants were as follows on the left side: class 1 (n = 7), class 2 (n = 1), and class 3 (n = 5); on the right side, they were as follows: class 1 (n = 6), class 2 (n = 2), and class 3 (n = 5). The volume of residual water, peak amplitude of SH EMG, and duration of SH EMG activity in the four head and mandibular positions, and the statistical results of the repeated measures analysis of variance are shown in Table 2. The statistical significance was observed with respect to each outcome variable. The intergroup comparisons of each outcome variable using Tukey’s test are shown in Table 3.

**Table 1:** Characteristics of the subjects.

Variables	Mean ± SD	Median (IQR [Range])
Age, year	34.0 ± 8.0	29 (28–40 [25–49])
Height, cm	157.7 ± 4.5	157.2 (155–160 [150–166])
Weight, kg	47.8 ± 5.6	50 (44–52 [39–56])
Body mass index, kg/m^2^	19.2 ± 1.6	19.5 (17.9–19.9 [16.7–22.2])
Overbite, mm	3.2 ± 2.1	3.1 (1.4–4.2 [0–7.7])
Overjet, mm	3.3 ± 1.6	3.2 (2.2–3.6 [0.6–6.7])
Mandibular advancement, mm	5.0 ± 1.8	4.9 (3.7–6.1 [1.6–8.6])
Maximum interincisor distance, mm	43.9 ± 4.6	43.0 (41.1–46.1 [37.6–52.8])

SD, Standard deviation; IQR, Inter-quartile range. Data are expressed as mean ± standard deviation and median (IQR [range]).

**Table 2:** Variables measured in the four head and mandibular positions and statistical analysis.

Variables	Position				F value	P value
	Neutral	Extension	Opening	Advancement		
Volume of residual water, ml	0.24 ± 0.37	1.11 ± 1.14	7.95 ± 2.61	4.73 ± 3.46	33.728	<0.001*
**Peak amplitude of SH EMG**						
Raw value, mV	0.34 ± 0.12	0.39 ± 0.14	0.26 ± 0.15	0.30 ± 0.13	3.783	0.019
Normalised value, %	100	123.5 ± 53.4	79.8 ± 42.5	96.0 ± 52.7	3.916	0.016
Duration of SH EMG activity, s.	1.34 ± 0.35	1.56 ± 0.33	1.23 ± 0.31	1.28 ± 0.36	4.095	0.013

Data are expressed as mean ± standard deviation. Neutral, Neutral position; Extension, Head extension position; Opening, Mouth opening position; Advancement, mandibular advancement position; SH EMG, Electromyogram of the suprahyoid muscles. *Data were corrected using the Greenhouse–Geisser method (Mauchly’s sphericity test was used to confirm the sphericity of the data; in cases in which the sphericity was violated, the data were corrected using the Greenhouse–Geisser method).

**Table 3:** Intergroup comparisons of the measured variables.

Variables	Positions		Difference	95% Confidence interval	P value
	(A)	(B)	(A)-(B)		
Volume of residual water, ml	Neutral	Extension	−0.87	−3.19 to 1.45	0.746
		Opening	−7.71	−10.03 to −5.38	<0.001
		Advancement	−4.49	−6.82 to −2.17	<0.001
	Extension	Opening	−6.84	−9.16 to −4.52	<0.001
		Advancement	−3.62	−5.95 to −1.30	0.001
	Opening	Advancement	3.22	0.89 to 5.54	0.004
Peak amplitude of SH EMG (Raw value), mV	Neutral	Extension	−0.04	−0.15 to 0.06	0.696
		Opening	0.08	−0.02 to 0.19	0.176
		Advancement	0.44	−0.06 to 0.15	0.691
	Extension	Opening	0.13	0.19 to 0.23	0.015
		Advancement	0.09	−0.20 to 0.19	0.144
	Opening	Advancement	−0.04	−0.15 to 0.07	0.756
Peak amplitude of SH EMG (Normalised value), %	Neutral	Extension	−23.5	−58.2 to 11.2	0.279
		Opening	20.2	−14.5 to 54.9	0.411
		Advancement	4.0	−30.7 to 38.8	0.989
	Extension	Opening	43.69	9.0 to 78.4	0.009
		Advancement	27.5	−7.2 to 62.3	0.144
	Opening	Advancement	−16.2	−50.9 to 18.6	0.598
**Duration of SH EMG activity, s**.					
	Neutral	Extension	−0.22	−0.45 to 0.05	0.145
		Opening	0.11	−0.17 to 0.38	0.716
		Advancement	0.06	−0.22 to 0.33	0.947
	Extension	Opening	0.33	0.06 to 0.60	0.013
		Advancement	0.28	0.00 to 0.55	0.045
	Opening	Advancement	−0.05	−0.32 to 0.22	0.955

The data were compared using Tukey’s test. Neutral, Neutral position; Extension, Head extension position; Opening, Mouth opening position; Advancement, mandibular advancement position; SH EMG, Electromyogram of the suprahyoid muscles.

### Volume of residual water

A greater volume of water remained in the mouth during the mandibular advancement and the mouth opening positions when compared with the neutral position. The volume of the residual water in the mouth during mandibular advancement was larger than that during the head extension position, but smaller with mouth opening.

### Peak amplitude of SH EMG

Although the peak amplitude of SH EMG was smaller with mandibular advancement than with head extension, there was no significant difference in EMG values during mandibular advancement when compared with the values obtained in the neutral position. The peak amplitude of SH EMG was also smaller with the mouth opening position than with the head extension position. Other significant differences were not observed.

### Duration of SH EMG activity

Although the duration of the SH EMG activity was shorter with mandibular advancement than with head extension, there was no significant difference in the EMG values with mandibular advancement when compared with that in the neutral position. The duration of the SH EMG activity was longer with head extension than that with mouth opening and mandibular advancement positions.

## Discussion

The swallowing ability in the mandibular advancement position is associated with greater impairment than that in the head extension position, but lesser than that in the mouth opening position. Although the swallowing test performed in this study is not an accurate reflection of the participants’ swallowing ability, the presence of high volumes of residual water appears to provide sufficient evidence of swallowing impairment. There was no significant difference in the EMG values obtained during the mandibular advancement position compared with that obtained during the neutral position. Thus, the swallowing ability is most impaired during the mouth-open position, which is usually required during dental treatment. Head extension^9^ or mandibular advancement^10^ is required when the airway obstruction is observed during the dental treatment under intravenous sedation. To the best of our knowledge, this is the first study to evaluate the impairing effect of the mandibular advancement, head extension, and mouth opening on the ability to swallow in the supine position.

In previous studies of swallowing ability, EMG^11,12^, manometry^13,14^, timing of swallowing^14^, radiography^13^, or fibreoptic endoscopic evaluation of swallowing^15^ was used. Radiography or manometry may be superior to electromyography; however, the insertion of a catheter can influence salivary secretion^16^. Moreover, radiography requires participants to be exposed to X-ray radiation. Therefore, in the present study we measured the volume of residual water and the EMG value to assess swallowing ability and avoid external factors and X-ray exposure.

In the present study, we investigated the swallowing ability in the supine position since it is the widely used position in most dental treatments. Although some influences of the body posture on swallowing were observed, swallowing was successfully performed in the supine position in previous studies^6,11,17,18^. Therefore, as in these previous studies, we considered the supine position with the neutral head position as the baseline^5^.

There are a few previous studies that examine the influence of mandibular advancement on the ability to swallow, likely because mandibular advancement is a non-physiological position. However, in this study, no significant differences were observed in the EMG values obtained during the mandibular advancement position compared with the neutral position. The difficulty of mouth closure during mandibular advancement might be associated with a decrease in the swallowing ability, as observed with mouth opening^5^. In general, swallowing requires anterior sealing between the tongue and the palate to make negative intra-oral pressure with the ‘mouth closed’ condition. However, this process does not always require active mouth closure^19^, and swallowing can be performed with the mouth open. Therefore, swallowing ability in the mandibular advancement position might be associated with more easily generating negative intra-oral pressure, as obvious differences were not observed in EMG values.

Patients with cognitive impairments, severe gag reflex, or severe dental phobia usually require intravenous d eep sedation during dental treatment. In these cases, the mandibular advancement or head extension is usually required because intravenous deep sedation is likely to induce airway obstruction. Dental staff are expected to maintain airway management, and to avoid the suction of intra-oral fluid in these circumstances. Therefore, when the patients fail to swallow the intra-oral fluids during the dental treatment, the cough reflex might be triggered. Although Ayuse *et al*.^20^ reported that mandibular advancement influences the coordination between respiration and non-nutritive swallowing, the extent of the impaired swallowing ability with the mandibular advancement position was not investigated. However, other studies have investigated the influence of mandibular advancement on airway management.

Mandibular advancement decreases nasal airway resistance during sedation^21^. Thus, mandibular advancement is often effective in releasing airway obstruction during dental treatment under intravenous sedation. In fact, the dose-dependent effect of mandibular advancement on the pharyngeal patency has been reported^22^. It has been described that a 25% advancement in position (of the maximum protrusion) made it possible to reduce the apnoea-hypopnea index in obstructive sleep apnoea syndrome (OSAS) patients^23^. Thus, like many studies on OSAS patients have reported, mandibular advancement has the advantage of providing airway protection without maximum advancement. The mechanism of this effect is explained by the improvements in the oropharyngeal and velopharyngeal airways, which are induced by mandibular advancement^24^. Despite the mechanism involved, it is important to be aware of the positive effect of mandibular advancement on airway protection, despite its negative effect on the swallowing ability.

In the present study, the impaired swallowing ability induced by the head extension was smaller than that induced by the mouth opening or mandibular advancement. This suggests that the head extension may serve as a better airway management position in terms of preserving the swallowing ability. However, it is important to consider that swallowing takes the longest in the head extension position. Previous studies also demonstrated that the head extension resulted in swallowing impairment^12,25^. Despite the association between swallowing impairment and the head extension position, the patient’s head is often extended during the dental treatment under intravenous sedation because head extension is found to be an effective technique for promoting airway protection. This method increases the maximum oropharyngeal airway size and decreases the closing pressure of the velopharynx and oropharynx^26^.

In the present study, the mouth opening position resulted in greater swallowing impairment than that from the head extension or mandibular advancement. Moreover, the mouth opening position easily induces upper airway obstruction^26,27^. Although the mouth-open position is usually required during the dental treatment, it impairs both the airway patency and the swallowing ability. Therefore, care should be taken during the dental treatment under intravenous sedation to prevent the upper airway obstruction and the cough reflex.

The angle class was not the same in all subjects; however, we considered the influence of each subject’s malocclusion on swallowing ability to be inconsequential, as those presenting with maxillary or mandibular prognathism, trismus, apertognathia, or swallowing impairment were excluded from this study. Additionally, in this study, ‘mouth opening’ was defined as an interincisal distance of 30 mm in all subjects. The level of discomfort associated with an interincisal distance of 30 mm varied for each subject owing to the differences in the interincisal distance during maximum mouth opening. A dental air turbine handpiece and other equipment of a similar size are routinely used during dental procedures. Therefore, in this study, we considered a fixed level of mouth opening to be desirable, as is necessary for dental procedures, regardless of the individual differences.

As mandibular advancement is non-physiological, as described above, conscious patients rarely undergo dental treatment in the mandibular advancement position. However, mandibular advancement is required during dental treatments under intravenous sedation, especially under deep sedation such as in patients with severe cognitive impairment, because the sedatives trigger airway obstruction. Moreover, aspiration and swallowing impairment increase with propofol sedation^15^. In these situations, our results could not completely explain the swallowing ability; however, they suggest that head extension might be a better method compared with mandibular advancement to improve the airway patency and to reduce the occurrence of cough reflex, despite the wakefulness of the subjects.

The present study had several limitations. First, voluntary swallowing is different from spontaneous swallowing^28^. This study investigated the voluntary swallowing and not spontaneous swallowing; yet voluntary swallowing is not always the one that occurs during most dental treatments. However, it is difficult to perform a spontaneous swallowing test under the conditions outlined by this study. Second, this study did not include intravenous sedation, because performing a swallowing test in a sedated patient with mandibular advancement poses the potential risk of asphyxiation. Third, this study had a small number of participants. Although sufficient data on the volume of residual water were obtained in this study, there were no significant differences in the EMG values in the mandibular advancement position when compared with that in the neutral position. In terms of obtaining a more accurate EMG assessment, a greater sample size may be required. However, ethical considerations make it difficult to perform this volunteer study with a larger number of participants. Finally, the study was limited by its use of a mandibular advancement appliance. Although a mandibular advancement appliance is not used during dental treatment in a clinical setting, this appliance was needed to reproduce the determined level of mandibular advancement in the present study.

In conclusion, the swallowing ability in the mandibular advancement position is associated with greater impairment compared with that in the head extension position but less impaired than in the mouth-open position. Therefore, the head extension may serve as a better airway management technique than mandibular advancement in terms of preserving the swallowing ability. Further study is, therefore, needed to elucidate the effects of sedatives on the swallowing ability during dental treatment under intravenous sedation. Moreover, studies with a larger sample size might be needed for the further interpretation of the EMG values.

## Data Availability

The data are not available for public access because of patient privacy concerns; however, these can be obtained from the corresponding author on reasonable request.
